# Vacuolating Cytotoxin A Triggers Mitophagy in *Helicobacter pylori*-Infected Human Gastric Epithelium Cells

**DOI:** 10.3389/fonc.2022.881829

**Published:** 2022-07-14

**Authors:** Li Wang, Juan Yi, Xiao-Yang Yin, Jin-Xia Hou, Jing Chen, Bei Xie, Gang Chen, Qun-Feng Wang, Li-Na Wang, Xiao-Yuan Wang, Jing Sun, Lei-Ming Huo, Tuan-Jie Che, Hu-Lai Wei

**Affiliations:** ^1^ Key Laboratory of Preclinical Study for New Drugs of Gansu Province, School of Basic Medical Sciences, Lanzhou University, Lanzhou, China; ^2^ Geriatrics Department, The Second Hospital of Lanzhou University, Lanzhou, China; ^3^ Neurosurgery Department, The First Hospital of Lanzhou University, Lanzhou, China; ^4^ Key Laboratory of Functional Genomics and Molecular Diagnosis of Gansu Province, Lanzhou Baiyuan Gene Technology Co., Ltd, Lanzhou, China

**Keywords:** *Helicobacter pylori*, GES-1 cells, autophagy, VacA, mitophagy, TOM complex, PGAM5, STOM

## Abstract

*Helicobacter pylori* (*H. pylori*)-derived vacuolating cytotoxin A (VacA) causes damage to various organelles, including mitochondria, and induces autophagy and cell death. However, it is unknown whether VacA-induced mitochondrial damage can develop into mitophagy. In this study, we found that *H. pylori*, *H. pylori* culture filtrate (HPCF), and VacA could activate autophagy in a gastric epithelial cell line (GES-1). VacA-caused mitochondrial depolarization retards the import of PINK1 into the damaged mitochondria and evokes mitophagy. And, among mass spectrometry (LC-MS/MS) identified 25 mitochondrial proteins bound with VacA, Tom20, Tom40, and Tom70, TOM complexes responsible for PINK1 import, were further identified as having the ability to bind VacA *in vitro* using pull-down assay, co-immunoprecipitation, and protein–protein docking. Additionally, we found that the cell membrane protein STOM and the mitochondrial inner membrane protein PGAM5 also interacted with VacA. These findings suggest that VacA captured by STOM forms endosomes to enter cells and target mitochondria. Then, VacA is transported into the mitochondrial membrane space through the TOM complexes, and PGAM5 aids in inserting VacA into the inner mitochondrial membrane to destroy the membrane potential, which promotes PINK1 accumulation and Parkin recruitment to induce mitophagy. This study helps us understand VacA entering mitochondria to induce the mitophagy process.

## Introduction

As a common clinically pathogenic microorganism, *Helicobacter pylori* (*H. pylori*), confirmed by Marshall and Warren in 1984 (1, 2), always attracts the attention of researchers. Various gastrointestinal diseases (such as gastritis, peptic ulcers, and even lymphoproliferative gastric lymphoma) (3–5) have been associated with this gram-negative, spiral microaerophilic bacterium. Since more than 50% of the population worldwide has almost been infected by this bacterium (6–8), it has been recognized as a class I carcinogen by the World Health Organization (WHO) in 1994. As one of the most critical toxin molecules secreted by *H. pylori*, vacuolating cytotoxin A (VacA) may be closely related to the above-mentioned diseases (8–10). Furthermore, studies have confirmed that internalized VacA can damage various organelles, including mitochondria (11). More detailed research has shown that VacA could specifically target mitochondria, whereby it is transported into the membrane space by the outer mitochondrial membrane and gets inserted into the membrane to form an anion channel to disrupt the mitochondrial membrane potential (MMP) and cause mitochondrial damage (12–14).

As the energy generator of cells, mitochondria participate in various biological processes, including programmed apoptosis and necrotic cell death (15, 16). Thus, mitochondrial quality and quantity must remain in homeostasis. Mitochondrial autophagy (mitophagy) is a response to clear damaged mitochondria (17–19). This is generally considered as the main mechanism of mitochondrial quality control. At present, three classical pathways mediating mitophagy have been studied: 1) PINK1/Parkin pathway; 2) the outer mitochondrial membrane (OMM) receptor-mediated pathway; and 3) inner mitochondrial membrane (IMM) receptor-mediated pathway (20). Although there are different specific pathways to remove damaged mitochondria, the general process is the same. The characteristics of the mitophagy process can be divided into four periods. 1) During the early stage, membrane permeability changes after mitochondrial damage, which leads to mitochondrial depolarization and activates mitochondrial autophagy-related proteins. 2) Damaged mitochondria are encapsulated by autophagosomes to form mitophagosomes. 3) After the fusion of metaphase mitophagosomes and lysosomes, mature mitolysosomes are formed. 4) Finally, mitochondria are degraded by lysosomes.

Increasing studies have demonstrated that *H. pylori* or *H. pylori* culture filtrate (HPCF) is associated with autophagy (21–23). In the battle for survival, *H. pylori* can subvert autophagy to establish its intracellular replication niche (21). VacA plays an indispensable role in this autophagy. Moreover, it has been confirmed that VacA is involved in various signaling pathways that induce autophagy (22), such as the mTORC1 pathway (24). Considering that VacA can enter host cells and induce mitochondrial dysfunction, it is still unclear whether damaged mitochondria will be selectively cleared by mitophagy.

In this study, we demonstrated that *H. pylori* or HPCF could promote apoptosis, inhibit proliferation, and induce autophagy by flow cytometry and western blot. In addition to showing the same ability as *H. pylori* or HPCF on GES-1 cells, VacA derived from *H. pylori* could also induce the decrease of MMP, which leads to mitophagy *in vitro*. Our data indicate that VacA might target mitochondria after binding to stomatin (STOM). The OMM proteins, such as Tom20, Tom40, and Tom70, transport VacA into the IMM with the help of phosphoglycerate mutase 5 (PGAM5) to destroy the MMP, inducing mitophagy *via* the PINK1/Parkin pathway. Overall, we believe that this study will be useful for obtaining a more comprehensive picture of the biological processes of mitophagy induced by VacA.

## Materials and Methods

### Bacterial Strain and Growth Conditions

The *Helicobacter pylori* (*H. pylori*) American Type Culture Collection (ATCC) 43504 (cagA^+^, vacA^+^) was purchased from the BeNa Culture Collection, Henan, China. *H. pylori* was grown on Columbia Blood Agar Base (Oxoid, Basingstoke, Hampshire, UK) plates containing 7% defibrillated sheep blood (njbianzhen, Nanjing, Jiangsu, China) in a humidified environment. Moreover, its liquid culture was carried out in BactoTM Brain Heart Infusion (BD Difco, Sparks, MD, USA) supplemented with 10% newborn calf serum (NBCS) (Gibco, Thermo Fisher Scientific, Waltham, MA, USA). Plates and liquid cultures were maintained at 37°C in the microaerophilic environment (5% O_2_, 10% CO_2_, and 85% N_2_). *Escherichia coli* TOP10 was used for plasmid amplification, and Rosetta-gami (DE3) pLysS (Fineyoungbio, Guangzhou, China) was used for protein expression. These were grown on Luria-Bertani (LB) agar (Solarbio, Beijing, China) or LB broth (Solarbio, Beijing, China) containing Ampicillin (100 μg/ml) at 37°C.

### Cell Culture

The human normal gastric mucosal epithelial cell line GES-1 was purchased from the Beijing Institute of Tumor Cells (Beijing, China). HeLa YFP-Parkin cells were received from Hanming Shen’s laboratory and were originally a kind gift from Dr. Richard Youle. Cells were grown in Dulbecco's modified Eagle medium (high glucose) (HyClone, Thermo Fisher Scientific, Waltham, MA, USA) supplemented with 10% NBCS in 5% CO_2_ atmosphere at 37°C. The same culture method was used for HeLa YFP-Parkin and GES-1 mRFP-GFP-hLC3B cells.

### Generation of LC3B-Overexpression GES-1 Cells

The recombinant pCDH-CMV-mRFP-GFP-hLC3B-EF1A-Puro plasmid (2 μg) (Fenghbio, Changsha, Hunan, China) was co-transfected with psPAX2 (1.5 μg) (#12259, Addgene, Cambridge, MA, USA) and pMD2.G (1 μg) (#12259, Addgene, Cambridge, MA, USA) into HEK293T cells in each well (6-well plate) to package infectious lentivirus. GES-1 cells were infected with concentrated lentivirus (with 6 μg/ml of polybrene added at the same time) for 24 h, followed by selection with 2 μg/ml puromycin. The puromycin-resistant GES-1 mRFP-GFP-hLC3B cells were confirmed for LC3B overexpression using an LC3B antibody (Cell Signaling Technology, Danvers, MA, USA).

### Cell Treatment

GES-1, GES-1 mRFP-GFP-hLC3B, or HeLa YFP-Parkin cells (5 × 10^4^ cells/ml) were seeded into 6-well or 96-well plates. The cells were allowed to attach overnight. After that, normal cell culture medium (negative control), normal cell culture medium containing *H. pylori* ATCC43504 (MOI = 100) (23, 25), and normal culture cell medium containing 50% (V/V) of culture filtrate (CF) for *H. pylori* culture, or normal cell culture medium containing 50% (V/V) of *H. pylori* culture filtrate (HPCF) were added and incubated for the indicated times. HPCF was obtained from *H. pylori* culture solution, which was filtered using a 0.22 μm filter. For p88 treating cells, acid-activated p88 was mixed with a normal cell culture medium and exposed to cells (negative control extract without p88 protein (pCold-GST).

### Plasmid Construction

For the recombinant VacA expression plasmid construction, the vacA sequence (amino acids 1–821 of the secreted mature VacA toxin) was cloned from *H. pylori* strain ATCC 43504 into pCold II. The Cetyltrimethylammonium Bromide (CTAB) (Solarbio, Beijing, China) method was used for extracting genomic DNA (gDNA). The TRIZOL (Invitrogen, Thermo Fisher Scientific, Waltham, MA, USA) method was used to extract RNA from HeLa cells to obtain cDNA for templating mitochondrial-related genes. These genes were cloned into pCold-GST. Two primers, p88F, 5’-CGGGATCCATGTTTTTCACAACCGTG-3’ and p88R, 5’-CGGAATTCTTAGTGGTGGTGGTGGTGGTGGTGGAGAGCGTAGTTAGCAG-3’ were used for polymerase chain reaction (PCR) amplification. Primers of other genes are listed in the supplementary materials (**Table S7**). All PCR products, pCold II or pCold-GST, were digested with BamHI and EcoRI (Thermo Fisher Scientific, Waltham, MA, USA) and ligated with a Takara DNA Ligation Kit (Takara Bio, Dalian, Liaoning, China).

VacA overexpression plasmid was constructed from plasmid pcDNA3.1-flag-HA (Fenghbio, Changsha, Hunan, China), which was digested with BamHI and XhoI (Thermo Fisher Scientific, Waltham, MA, USA). Plasmid pcDNA3.1-myc-His (Fenghbio, Changsha, Hunan, China) was digested with BamHI and EcoRI (Thermo Fisher Scientific, Waltham, MA, USA) to overexpress Tom20, Tom40, Tom70, STOM, and PGAM5. The primers for these genes are listed in the supplementary materials (**Table S8**).

### Expression and Purification of Recombinant Protein

The recombinant plasmid, which has been transformed into TOP10 for amplification and confirmed by sequencing, was transformed into Rosetta-gami (DE3) pLysS chemically competent cells. The transformants were grown on LB agar (containing 100 μg/ml ampicillin and 34 μg/ml chloramphenicol) to form a single colony, which was then inoculated into Terrific Broth (TB) (containing 100 μg/ml ampicillin and 34 μg/ml chloramphenicol) and grown at 37°C overnight with shaking at 220 rpm. Incubation was followed by diluting the samples (1:100) into TB (containing 100 μg/ml ampicillin and 34 μg/ml chloramphenicol). These cultures were shaken at 200 rpm and 37°C for about 4 h to reach an OD600 between 0.6 and 0.8. Samples were cultured further for 30 min at 15°C with shaking at 150 rpm. The cells adapted to the low culture temperatures and were finally induced with isopropyl-β-D-thiogalactopyranoside (IPTG) at a concentration of 0.02 mM (other proteins were induced at 0.1 mM). Samples were further incubated at 15°C and shaken at 150 rpm for 24 h. Cultures were then centrifuged at 8,000 rpm for 10 min at 4°C, the supernatant was discarded, and the bacterial precipitation was collected and stored at −20°C until use.

*Escherichia coli* (*E. coli*) precipitation containing VacA was resuspended in a lysis buffer (50 mM Tris (pH 7.4), 500 mM NaCl, 25 mM imidazole, 0.1% Triton X-100, and 10% glycerol). *E. coli* precipitation containing mitochondrial-related proteins was resuspended in a lysis buffer (50 mM Tris (pH 7.4), 150 mM NaCl, 1 mM dithiothreitol (DTT), 0.1% Triton X-100, and 10% glycerol). The solutions underwent high-pressure homogenization (700–800 bar) at 4°C until transparent. After the transparent solution was centrifuged at 8,000 rpm for 20 min at 4°C, the supernatant was filtered. The *E. coli*-soluble extracts containing VacA were purified using HisSep Ni-NTA agarose resin (Yeasen, Shanghai, China) and were used to pack the gravity column according to the instructions of the manufacturer. The *E. coli*-soluble extracts containing mitochondrial-related proteins were purified using GSTSep Glutathione agarose resin (Yeasen, Shanghai, China) according to the instructions of the manufacturer. All purified proteins or cell lysates were quantified using the bicinchoninic acid (BCA) method. All purified recombinant VacA (p88) was acid activated by adding 1 M hydrochloric acid (HCl) to lower the pH to 3 for 30 min at 37°C. The same volume of 1 M sodium hydroxide (NaOH) was added to raise the pH to 7.0 for use.

### SDS-PAGE and Western Blot

*E. coli* debris or extracts containing our target proteins were separated using sodium dodecyl sulfate-polyacrylamide gel electrophoresis (SDS-PAGE). Coomassie brilliant blue staining (0.1% Coomassie brilliant blue R-250, 50% methanol, and 10% glacial acetic acid) and decolorization (5% methanol and 7.5% glacial acetic acid) were used to visually test the protein expression and its relative amount. For western blots, *E. coli* extracts (lysed in phosphate-buffered saline (PBS), pH 7.2) or cell lysates (lysed in SDS sample buffer (63 mM Tris–HCl, 10% glycerol, and 2% SDS)) were separated using SDS-PAGE and then transferred onto polyvinylidene fluoride (PVDF) membranes (Merck Millipore, Burlington, MA, USA). After blocking with 5% skim milk, the membranes were immunoblotted with the corresponding primary and secondary antibodies. The primary antibodies used in our study were as follows: VacA (Santa Cruz Biotechnology, Santa Cruz, CA, USA), LC3B (Cell Signaling Technology, Danvers, MA, USA), PINK1 (Cell Signaling Technology, Danvers, MA, USA), Parkin (Cell Signaling Technology, Danvers, MA, USA), Tom20 (Cell Signaling Technology, Danvers, MA, USA), phospho-ubiquitin (Merck Sigma-Aldrich, Burlington, MA, USA), Tim23 (BD Biosciences, Franklin Lakes, NJ, USA), GST (Abbkine, Wuhan, China), Flag (GeneTex, Irvine, CA, USA), c-Myc (GeneTex, Irvine, CA, USA), and β-actin (Servicebio, Wuhan, China). Images of protein bands were used to perform relative quantitative analysis using ImageJ (version 1.52a; National Institutes of Health, Bethesda, MA, USA) along with Photoshop (version 19.0; Adobe, San Jose, CA, USA).

### Vacuolating Assay

To induce vacuole formation, various concentrations of purified acid-activated p88 were mixed with the culture medium and 10 mM ammonium chloride to a final volume of 100 μl per well (26). After the normal culture medium was discarded, this mixture medium was added to GES-1 cells for 4 h at 37°C. Cell vacuolation images were captured using an inverted optical microscope. The media mixtures were replaced with 50 μl of 0.5% neutral red per well and allowed to stain for 5 min at 37°C. The cells were washed thrice with PBS (100 μl). After that, 100 μl of acidified alcohol (50% alcohol and 1% acetic acid) was added to extract the neutral red from the cells at 37°C for 5 min. Lastly, these decolorizing solutions were transferred to a clean 96-well plate to determine the optical density (OD) at 540 nm using a Microplate Reader (Agilent, Santa Clara, CA, USA) (27).

### Mitochondrial Membrane Potential (MMP) Analysis and Flow Cytometry

For the mitochondrial membrane potential (MMP) analysis, HeLa YFP-Parkin and GES-1 cells were seeded into 96-well (for MMP observation) or 6-well plates (for flow cytometry detection) overnight before each experiment. After that, the medium was replaced with purified acid-activated p88 (12 μg/ml) mixed with the culture medium (100 μl/well for the 96-well plate or 1 ml/well for the 6-well plate), and cells were incubated for 1 h at 37°C. Reduced MMP was observed and images were obtained using an inverted fluorescence microscope. All GES-1 cells in the 6-well plate were washed twice using cold PBS, followed by digestion and centrifugation. GES-1 cells were then collected and the MMP was determined using a JC-1 kit (Solarbio, Beijing, China), following the instructions of the manufacturer. Briefly, cell precipitation was resuspended in 0.5 ml of JC-1 staining work solution and incubated at 37°C for 20 min. After centrifugation (600 rpm for 4 min at 4°C), the cell precipitation was washed twice using 1 ml of JC-1 staining buffer, followed by a second centrifugation step (600×*g* for 4 min at 4°C). After that, the cell precipitation was resuspended using 0.5 ml of JC-1 staining buffer, and the samples were interrogated using flow cytometry (NovoCyte, Agilent, Santa Clara, CA, USA). The proliferation (Beyotime, Shanghai, China) and apoptosis (BD Biosciences, Franklin Lakes, NJ, USA) of GES-1 cells were detected using flow cytometry following the instructions of the manufacturer.

### His Pull-Down and GST Pull-Down

For His pull-down, GES-1 cells were washed twice with cold PBS and lysed with 250 μl lysis buffer (50 mM Tris–HCl, 500 mM NaCl, 25 mM imidazole, 0.1% Triton X-100, 10% glycerol, and 2% SDS) containing protease inhibitors at 4°C for 30 min. Regarding the ice bath, the viscous sample was crushed using an ultrasonic crusher for 30 s. After adding 50 μl of HisSep Ni-NTA agarose resin (Yeasen, Shanghai, China) and 200 μl of purified acid-activated (or not) p88, the mixture was incubated with shaking at 80 rpm overnight at 4°C. Samples were then centrifuged at 750 rpm for 5 min at 4°C. The mixture was washed thrice with the lysis buffer, followed by a second centrifugation step (same conditions). The elution buffer (50 mM Tris–HCl, 500 mM NaCl, 150 mM imidazole, 0.1% Triton X-100, 10% glycerol, pH 7.4) was added to resuspend the mixture, followed by centrifugation (750 rpm for 5 min at 4°C) to bind the proteins to the agarose resin. The obtained supernatant was subjected to SDS-PAGE, and the gel was sent to Hangzhou Ptm-Biolab Biotechnology Co., Ltd (Hangzhou, Zhejiang, China) for LC-MS/MS analysis. For the GST pull-down, 50 μl of agarose resin (Yeasen, Shanghai, China), combined with recombinant mitochondrial associated proteins, was transferred into a clean microcentrifuge tube. Simultaneously, 200 μl of purified acid-activated p88 was added and incubated with shaking at 80 rpm overnight at 4°C. The samples were centrifuged (750 rpm, 5 min, 4°C), and the mixture was washed thrice with wash buffer (50 mM Tris–HCl, 150 mM NaCl, pH 7.4). Approximately 90 μl of elution buffer (50 mM Tris–HCl, 150 mM NaCl, 150 mM reduced glutathione, pH 7.4) was added to resuspend the mixture, and the samples were centrifuged again (same conditions). The obtained supernatant was subjected to a western blot.

### GO Terms and KEGG Pathway Enrichment

The proteins identified by LC-MS/MS were introduced into the DAVID Bioinformatics Resources 6.8 (28, 29) for enrichment analysis of Gene Ontology (GO) terms and Kyoto Encyclopedia of Genes and Genomes (KEGG) pathways. The ggplot2 package (version 3.3.3; H. Wickham. ggplot2: Elegant Graphics for Data Analysis. Springer-Verlag, NYC, USA) in R (version 4.0.3; R Core Team (2021). R: A language and environment for statistical computing. The R Foundation for Statistical Computing, Vienna, Austria) was used to visualize the analysis results.

### Mitochondrial Fractionation

GES-1 cells were grown in culture flasks until 90% confluency, then washed once with cold PBS. After digestion using trypsin, cells were collected by centrifugation (800 rpm, 5 min). Cells were washed with cold PBS and resuspended in extraction buffer (20 mM Hepes-KOH, 1.5 mM MgCl_2_, 1 mM EDTA, 250 mM sucrose, 10 mM KCl, 1 mM dithiothreitol, and 0.1 mM PMSF) (30). A Teflon-glass homogenizer was used to homogenize the cellular suspension. After centrifugation (750×*g* for 10 min at 4°C), the supernatant was collected and the precipitate was added to the extraction buffer again. The supernatants obtained from the two were pooled and centrifuged at 10,000×*g* for 15 min at 4°C. The pellets (crude mitochondria) were resuspended in the import buffer (3% W/V fatty acid-free bovine serum albumin (BSA), 80 mM KCl, 250 mM sucrose, 5 mM MgCl_2_, 10 mM MOPS-KOH, 2 mM K_2_HPO_4_, and 10 μM ATP) for 30 min at 4°C (31).

### Quantification of Mitochondrial DNA Content

GES-1or HeLa YFP-Parkin cells (5 × 10^4^ cells/ml) were seeded into a 6-well plate. After 24 h, cells were treated with p88 (12 μg/ml) for 1 h. After that, cells were washed twice with PBS, and the TRIZOL (Invitrogen, Thermo Fisher Scientific, Waltham, MA, USA) method was used to extract total RNA from cells for RT-qPCR. RT-qPCR was carried out according to the instructions of the manufacturer (Yeasen, Shanghai, China). MtDNA (mitochondrial 16S rRNA gene) and nuclear DNA (nDNA, β2-microglobulin gene) were quantified by quantitative PCR (ROCGENE, Beijing, China) using the Archimed X5 real-time PCR detection system (ROCGENE, Beijing, China). The primers used were: mtDNA (F, 5′-ACCTTACTACCAGACAACCTTAG-3′, and R, 5′-ACATAGACGGGTGTGCTC-3′) and nDNA (F, 5′-TTCATCCATCCGACATTGA-3′, and R, 5′-ACGGCAGGCATACTCATCT-3′). Mitochondrial DNA content was calculated using 2 × 2 ^(nDNAC^_T_^-mtDNAC^_T_^)^ (32).

### Immunoprecipitation Assay

After being transfected with overexpression plasmid pcDNA3.1-flag-HA-p88 or pcDNA3.1-Tom20-myc-His (Tom40, Tom70, STOM, and PGAM5) for 48 h, HEK293T cells were collected and lysed with IP lysis buffer. Lysed cells were incubated with the antibody Flag (GTX629631, GeneTex, Irvine, CA, USA) overnight at 4°C. Protein A/G beads (Yeasen, Shanghai, China) were washed with IP lysis buffer. After antibody binding, p88 or Tom20 was co-immunoprecipitated with agarose beads for 6 h at 4°C. The interaction was detected using Western blotting.

### Protease K Protection Assay

The protease K protection assay was performed as described previously (33). Briefly, after the isolated mitochondria were treated with p88, import buffer was used to wash the mitochondria three times. Samples were centrifuged (10,000×*g* for 15 min at 4°C) and finally resuspended in the import buffer (without protease inhibitor). When digesting the outer mitochondrial membrane, 50 μg/ml protease K (Solarbio, Beijing, China) was added and incubated on ice for 30 min. The digestion was terminated with a protease inhibitor. To facilitate digestion of mitochondrial intima and matrix, 0.3% Triton X-100 was added. Tom20 and p88 were detected using a Western blot.

### Immunofluorescence Assay

HeLa YFP-Parkin cells were inoculated into a 6-well plate placed with sterile coverslips. After 24 h, cells were treated with p88 (12 μg/ml), coverslips were removed, cells were washed twice with PBS, and 500 μl of 4% paraformaldehyde (Solarbio, Beijing, China) was added to fix cells for 20 min. PBS was used to wash the cells (three washes at 5 min per wash), followed by permeabilization with 0.1% Triton X-100 (diluted by PBS) for 20 min. After blocking with 1% BSA (dissolved in Tris Buffered Saline with Tween 20 (TBST)) for 1 h or one night at 4°C, cells were incubated with primary antibodies (Tom20, #42406, Cell Signaling Technology, Danvers, MA, USA) and secondary antibodies (Cy3 conjugated Goat Anti-Rabbit IgG, GB21303, Servicebio, Wuhan, China). After three washes with PBS, coverslips were placed on a glass slide with one drop of DAPI and then sealed. Samples were imaged using a fluorescence microscope (Olympus AX80, Tokyo, Japan) with a 100× oil lens.

### Transmission Electron Microscopy (TEM)

*H. pylori* or HPCF co-cultured GES-1 cells were fixed in glutaraldehyde (Solarbio, Beijing, China), postfixed in osmium tetraoxide, dehydrated in ethanol and acetone, and embedded in acetone and embedding solution. After curing, sectioning (~70 nm), and uranyl acetate-lead citrate double staining, a Tecnai G2 Spirit transmission electron microscope (TEM) (FEI, Hillsboro, OR, USA) was used to obtain digital images.

### Protein–Protein Docking

Biovia Discovery Studio Client (version 19.1.0; BIOVIA, Dassault Systèmes, San Diego, CA, USA) was used to perform protein–protein docking for these proteins: STOM (PDB ID: 4FVF), PGAM5 (PDB ID: 3MXO), Tom20 (AlphaFold DB ID: Q15388), Tom40 (PDB ID: 7CP9), Tom70 (PDB ID: 7DHG), and VacA (PDB ID: 6ODY).

### Statistical Analysis

All statistical analyses were performed using GraphPad Prism software (version 9.00; GraphPad Software Inc., San Diego, CA, USA). A two-tailed Student’s *t*-test was used to determine the significance of the difference between the two groups, followed by the Shapiro–Wilk test (for the normal (Gaussian) distribution test) and the F-test. To assess the difference between treatment groups and their controls, one-way ANOVA with Dunnett’s tests was used. A *p*-value of <0.05 was considered statistically significant. All the error bars indicate the standard deviation.

## Results

### *H. Pylori* Infection Promotes Apoptosis and Inhibits Proliferation in Gastric Mucosal Epithelial GES-1 Cells

**Figure 1:**
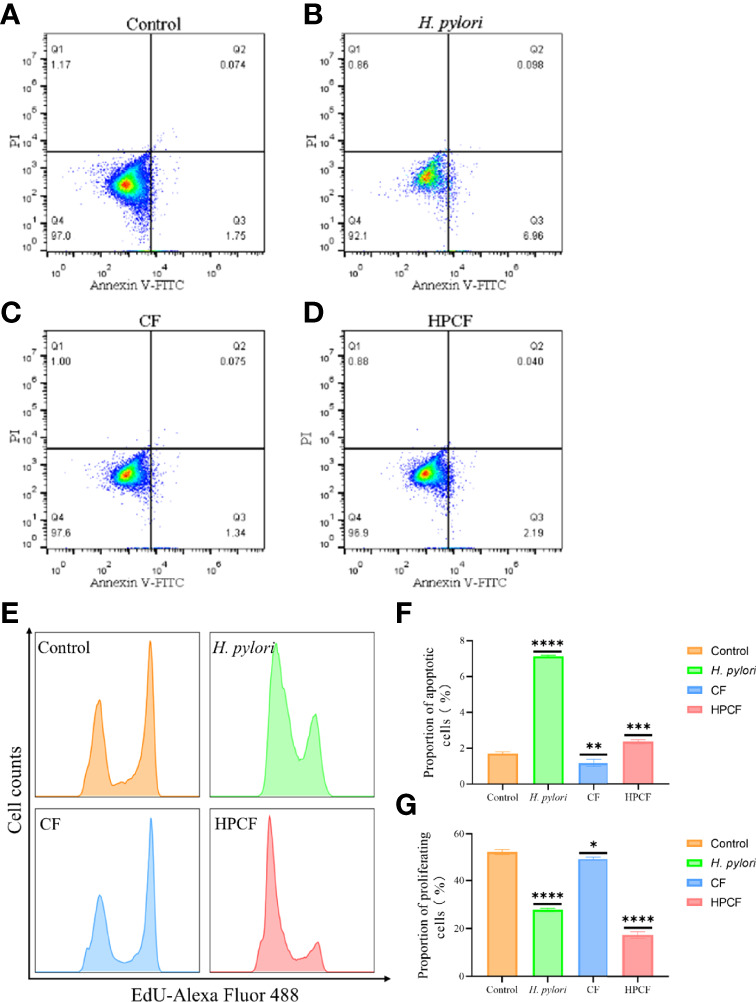
*H. pylori* or HPCF inhibit proliferation and induce apoptosis in GES-1 cells. Representative apoptosis photographs of GES-1 cells treated with normal cell culture medium **(A)**, *H. pylori* **(B)**, CF **(C)**, or HPCF **(D)** for 24 h using flow cytometry. The EdU-Alexa Fluor 488 cell count of GES-1 cells treated with these factors was detected by flow cytometry too **(E)**. Quantitative analysis of apoptosis (Q2 + Q3) **(F)** and proliferation **(G)** of GES-1 cells treated with *H. pylori*, CF, or HPCF was performed. The asterisks indicate significant differences from the control. n = 3. *p <0.05, **p <0.01, ***p <0.001, ****p <0.0001.

To clarify the effect of *Helicobacter pylori* (*H. pylori*) or *H. pylori* culture filtrate (HPCF) on cell apoptosis and proliferation of GES-1 cells, we co-cultured GES-1 cells with *H. pylori* or HPCF for 1 to 24 h followed by flow cytometry and cytomorphology detection. Apoptosis rate analysis showed a higher percentage of apoptotic GES-1 cells in *H. pylori* and HPCF than in control (**Figures 1B, D, F**), and no apoptosis appeared for less than 12 h (**Figure S1**). At the same time, the culture filtrate (CF) also showed a weak promoting apoptosis effect (**Figures 1C, F**). Decreased proliferation rates in *H. pylori*- or HPCF-treated GES-1 cells were also observed (**Figures 1E, G**).

Taken together, our data indicate that *H. pylori* or HPCF could promote apoptosis and inhibit proliferation in GES-1 cells.

### *H. Pylori* Infection Led to Autophagy/Mitophagy of GES−1 Cells

**Figure 2:**
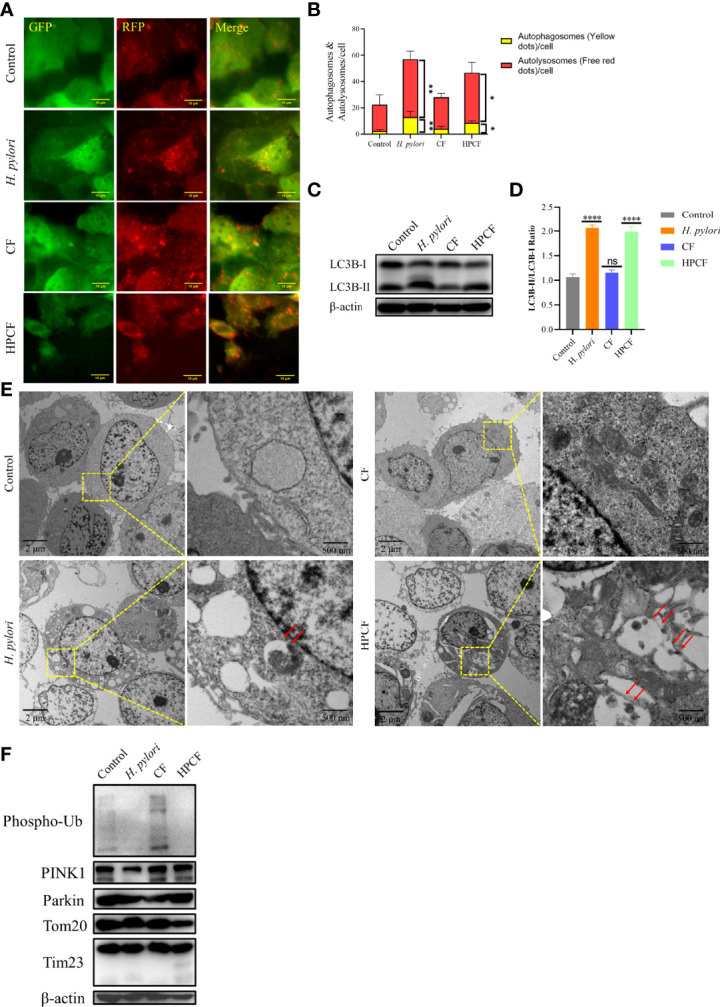
*H. pylori* or HPCF induced autophagy/mitophagy in GES−1 Cells. GES-1 mRFP-GFP-hLC3B cells were challenged with *H. pylori*, CF, or HPCF for 24 h, and the representative fluorescence images (Scale bar, 10 μm) of autophagosomes (yellow dots) and autolysosomes (red dots) using the tandem mRFP-GFP-hLC3B fusion protein assay **(A)**. The autophagy flux was evaluated by the ratio of red dots to yellow dots **(B)**. The conversion of LC3B-I to LC3B-II was detected using western blot **(C)**, and the level of change was quantified **(D)**. Representative images (Scale bars, 2 μm and 500 nm) of autophagosomes (indicated by red arrows) in GES-1 cells using the transmission electron microscope (TEM) **(E)**. The expression of mitophagy-related proteins was measured by western blot **(F)**. The asterisks indicate significant differences from the control. n = 3. *p <0.05, **p <0.01, ****p <0.0001. ns, no significance.

Previous reports that *H. pylori* or HPCF can induce autophagy led us to assess the occurrence of autophagy in GES-1 cells treated with these two factors for 24 h. To observe autophagy more conveniently, we first overexpressed pCDH-CMV-mRFP-GFP-hLC3B-EF1A-Puro in GES-1 cells (**Figure 2A**). In *H. pylori*-treated or HPCF-treated GES-1 cells, the red puncta were more numerous than the yellow puncta, which was in contrast with control or CF-treated GES-1 cells (**Figure 2B**). Meanwhile, the expression of an important indicator of autophagy, LC3B-II in GES-1 cells treated with *H. pylori* or HPCF was higher than that of the control and CF in GES-1 cells (**Figures 2C, D**). After being challenged by *H. pylori*, CF, or HPCF for 24 h, typical autophagosomes were formed (**Figure 2E**).

Having shown that *H. pylori* or HPCF can induce autophagy, we thus speculated that it is PINK1/Parkin-dependent mitophagy. Subsequently, we evaluated the expression of mitophagy-related proteins in those cells. The expression of PINK1 in *H. pylori*-treated GES-1 cells decreased, while it seemed to increase slightly in HPCF-treated GES-1 cells, compared with that of the control (**Figure 2F**). And, the expression of Phos-pho-Ubiquitin (Phospho-Ub) in both *H. pylori*-treated and HPCF-treated GES-1 cells was inhibited. Meanwhile, two mitochondrial-related membrane proteins (Tom20 and Tim23) decreased in HPCF-treated GES-1 cells.

These data indicate that *H. pylori* or HPCF could induce autophagy and change the expression levels of mitophagy-related proteins.

### VacA Induces Vacuolar Degeneration, Promotes Apoptosis, and Inhibits Proliferation in GES−1 Cells

**Figure 3:**
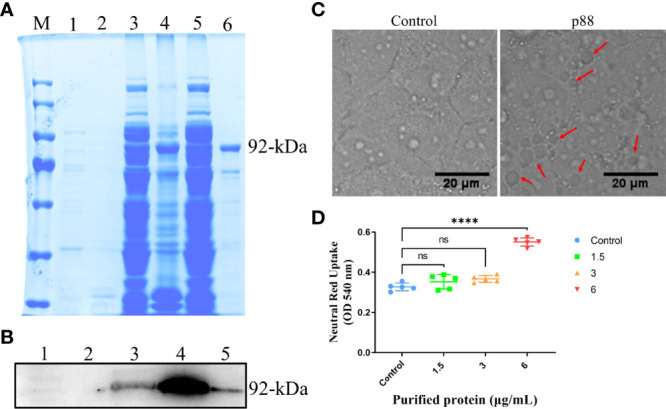
The expression and activity verification of p88. The *Escherichia coli* (*E. coli*) extracts containing recombinant VacA (p88) were analyzed using 8% sodium dodecyl sulfate-polyacrylamide gel electrophoresis (SDS-PAGE), and stained with coomassie brilliant blue. M, marker; 1, soluble fraction before induction; 2, insoluble fraction before induction; 3, soluble fraction after induction; 4, insoluble fraction after induction; 5, flow-through; 6, elution. The band in lane 6 labeled with 92-kDa indicates the purified p88 **(A)**. p88 was immunoblotted with a VacA antibody. 1, soluble fraction before induction; 2, insoluble fraction before induction; 3, soluble fraction after induction; 4, insoluble fraction after induction; 6, elution. The band in lane 5 labeled with 92-kDa indicates the purified p88 **(B)**. Representative images (Scale bar, 20 μm) of vacuoles (indicated by the red arrows) in GES-1 cells treated with p88 (6 μg/ml) for 4 h **(C)**. The vacuolating activity of different concentrations of p88 was measured by a neutral red uptake assay **(D)**. The asterisks indicate significant differences from the control. n = 5. ****p <0.0001. ns, no significance.

Among the numerous virulence factors derived from *H. pylori*, VacA, which has been widely studied regarding its effects on mitochondria and autophagy, was first considered. To study the exact regulation of the mature secreted ~88 kDa VacA on autophagy or mitophagy in GES-1 cells, we expressed it (~92-kDa) *in vitro*. Both methods of sodium dodecyl sulfate-polyacrylamide gel electrophoresis (SDS-PAGE) (**Figure 3A**) and western blot (**Figure 3B**) confirmed the successful expression of VacA (p88). VacA damages lysosomes and the endoplasmic reticulum of cells by targeting a specific receptor, leading to extensive vacuolar degeneration. The treatment of p88 on GES-1 cells increased the formation of vacuoles (**Figure 3C**, **Figure S4**). As p88 concentration increased, neutral red absorbance also increased, indicating a stronger vacuole activity (**Figure 3D**).

**Figure 4:**
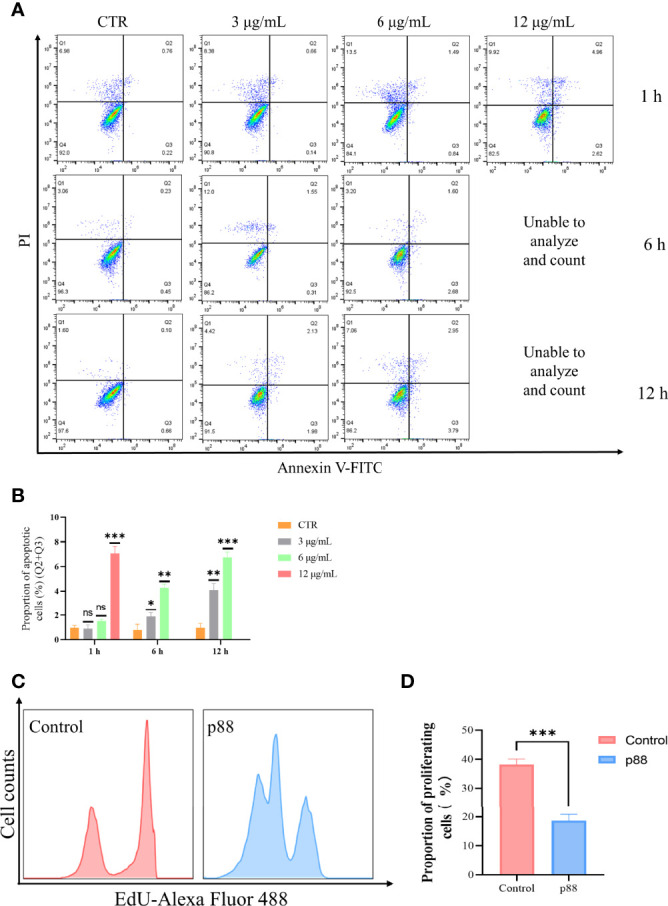
p88 induces apoptosis and inhibits proliferation in GES-1 cells. Representative apoptosis photographs of p88-treated GES-1 cells by flow cytometry **(A)**. Quantitative analysis of apoptosis (Q2 + Q3) **(B)**. The EdU-Alexa Fluor 488 cell count of 12 μg/ml p88-treated GES-1 cells was detected by flow cytometry **(C)**. Quantitative analysis of proliferation p88-treated GES-1 cells **(D)**. The asterisks indicate significant differences from the control. n = 3. *p <0.05, **p <0.01, ***p <0.001. ns, no significance.

Flow cytometry analysis showed that a high concentration of 12 μg/ml of p88 treatment mainly evoked GES-1 cells to show a certain degree of necrosis-like death, and after continuous treatment for more than 6 h, almost all the cells emerged dying or dead, which was presumed to be the aftereffect of efficient autophagy caused by the high concentration of p88 (VacA) (34). At lower concentrations of 1.5 to 6 μg/ml of p88 treatment for 1 h to 12 h, the apoptosis of GES-1 cells increased with the treatment time and concentration, and the obvious apoptosis appeared at 6 h and 12 h (**Figures 4A, B**, **S2**). To correspond to apoptosis enhancement, a lower proliferation rate existed in p88-treated GES-1 cells (**Figures 4C, D**).

These results suggest that p88 (VacA) expressed *in vitro* has vacuolating activity, which can promote apoptosis and inhibit proliferation in GES-1 cells.

### VacA Inducing the Depolarization of Mitochondria Develops Into Mitophagy of PINK1/Parkin Pathway in GES-1 Cells

**Figure 5:**
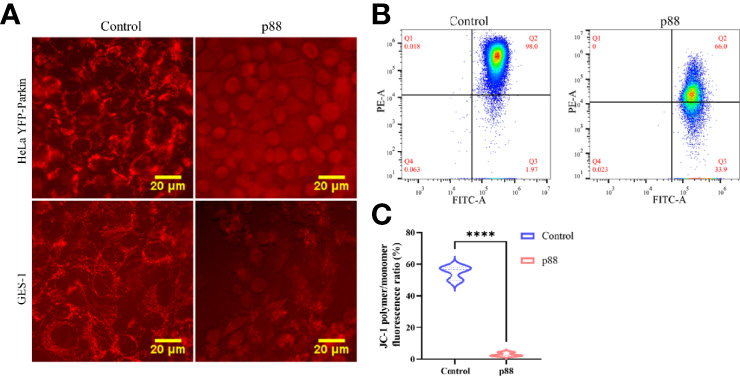
p88 induces the decrease of the mitochondrial membrane potential (MMP) in GES-1 cells. Representative fluorescence photographs (Scale bar, 20 μm) of the MMP in HeLa YFP-Parkin and GES-1 cells treated with p88 (12 μg/ml) for 1 h **(A)**. Representative the MMP photographs of p88-treated GES-1 cells were detected by flow cytometry with JC-1 **(B)**. Quantitative analysis of the MMP level in GES-1 cells treated with p88 was performed by the fluorescence ratio of JC-1 polymer/monomer **(C)**. n = 3. ****p <0.0001.

Considering that a high concentration of VacA can efficiently induce Parkin translocation of the cells (**Figure S3**), 12 μg/ml of p88 was selected to treat the cells in the autophagy-related study (34). According to previous reports that VacA entering the cell will target mitochondria and disrupt the mitochondrial membrane potential (MMP), we assessed the MMP of HeLa YFP-Parkin and GES-1 cells treated with p88 (12 μg/ml) for 1 h. A weakening of red fluorescence intensity was observed (**Figure 5A**). The quantitative analysis of the MMP level of p88-treated GES-1 cells also showed a significant decrease (**Figures 5B, C**). Yet decreased MMP is an indicator of mitophagy commonly.

**Figure 6:**
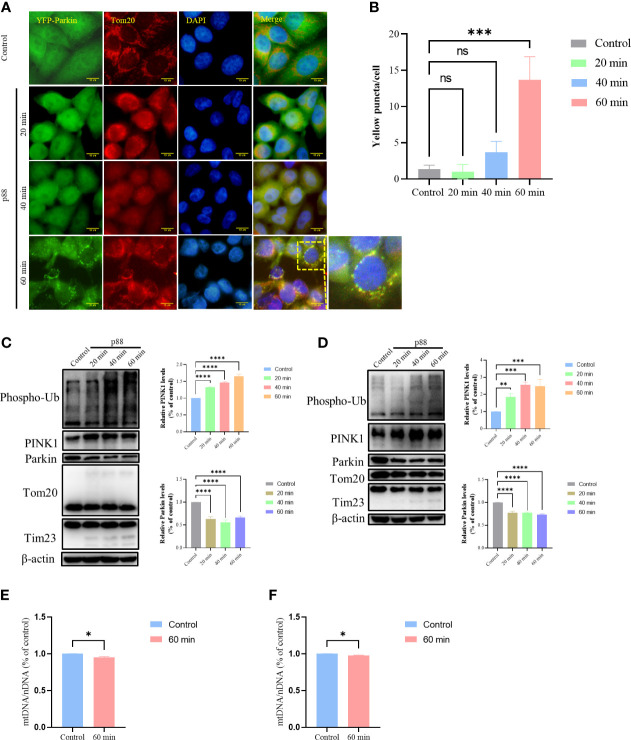
p88 induces mitophagy by PINK1/Parkin pathway in GES-1 cells. Representative fluorescence photographs (Scale bar, 10 μm) of mitophagy in HeLa YFP-Parkin cells treated with p88 (12 μg/ml) for 1 h **(A)**. The level of mitophagy was evaluated by the number of yellow puncta **(B)**. The asterisks indicate significant differences from the control. n = 3. ***p <0.001. ns, no significance. The expression levels of mitophagy-related proteins in HeLa YFP-Parkin **(C)** and GES-1 **(D)** cells were measured by western blot, and the level of change (PINK1 and Parkin) was quantified. After being treated with p88 (12 μg/ml) for 1 h, HeLa YFP-Parkin **(E)** and GES-1 **(F)** cells were detected with relative mitochondrial DNA content (mtDNA/nDNA) by RT-qPCR. The asterisks indicate significant differences from the control. n = 3. *p <0.05, **p <0.01, ***p <0.001, ****p <0.0001.

Having demonstrated that p88 induced a decrease in MMP in GES-1 cells, we investigated whether the damaged mitochondria would be selectively cleared by mitophagy. With the extension of time, p88 induced numerous puncta at 1 h (**Figures 6A, B**, **S3**) in HeLa YFP-Parkin cells. The Western blotting of mitophagy-related protein expression levels showed significant increases in Phospho-Ub and PINK1 in HeLa YFP-Parkin (**Figure 6C**) and GES-1 cells (**Figure 6D**) treated with p88, whereas the decreases in Parkin, Tom20, and Tim23 were not significant. Thus, the PINK1/Parkin pathway participates in VacA-induced mitophagy in GES-1 cells. Compared with the control, the content of mitochondrial DNA in HeLa YFP-Parkin (**Figure 6E**) and GES-1 (**Figure 6F**) cells treated with p88 for 1 h decreased.

### Interaction of VacA With Mitochondrial Membrane Proteins Mediated Activation of PINK1/Parkin-Dependent Mitophagy

**Figure 7:**
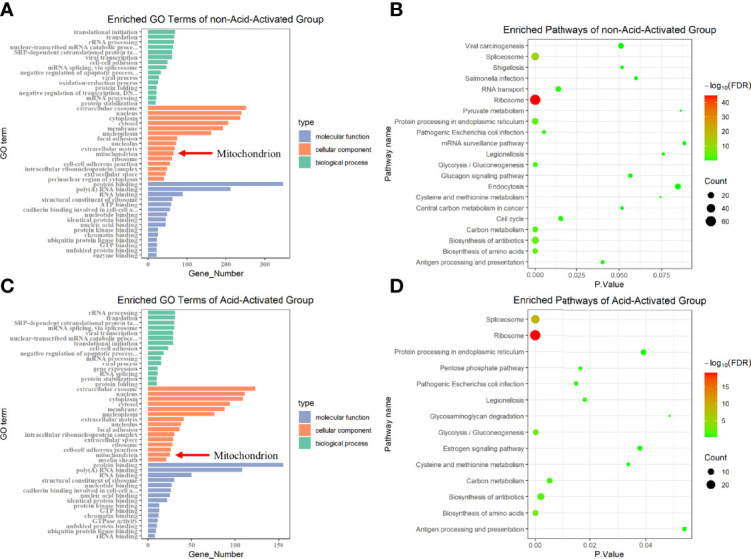
GO terms and KEGG pathway enrichment. Protein identification results of not-acid-activated and acid-activated groups were analyzed using DAVID Bioinformatics Re-sources and visualized using R. The Gene Ontology (GO) enrichment analysis was displayed in the form of bar graphs **(A, C)**, and the bubble graphs showed the corresponding enriched pathways **(B, D)**.

VacA has been reported to destroy MMP by forming anion channels in the mitochondrial inner membrane. Furthermore, we have demonstrated that the decrease of MMP induced PINK1/Parkin-dependent mitophagy in GES-1 cells. We next asked which proteins are involved in the insertion of VacA into the mitochondrial inner membrane. To accurately identify the proteins interacting with activated VacA, His pull-down and liquid chromatography-tandem mass spectrometry (LC-MS/MS) were performed (**Tables S1, S2**). Both cellular component (GO terms) enrichment of non-acid-activated and acid-activated groups indicated that mitochondria were involved (**Figures 7A, C**). Bubble plots of pathway enrichment showed that the pathway of ‘protein processing in endoplasmic reticulum’ played an important role in non-acid-activated and acid-activated groups (**Figures 7B, D**). Detailed enrichment results were listed in the supplementary materials (**Tables S3–S6**).

**Figure 8:**
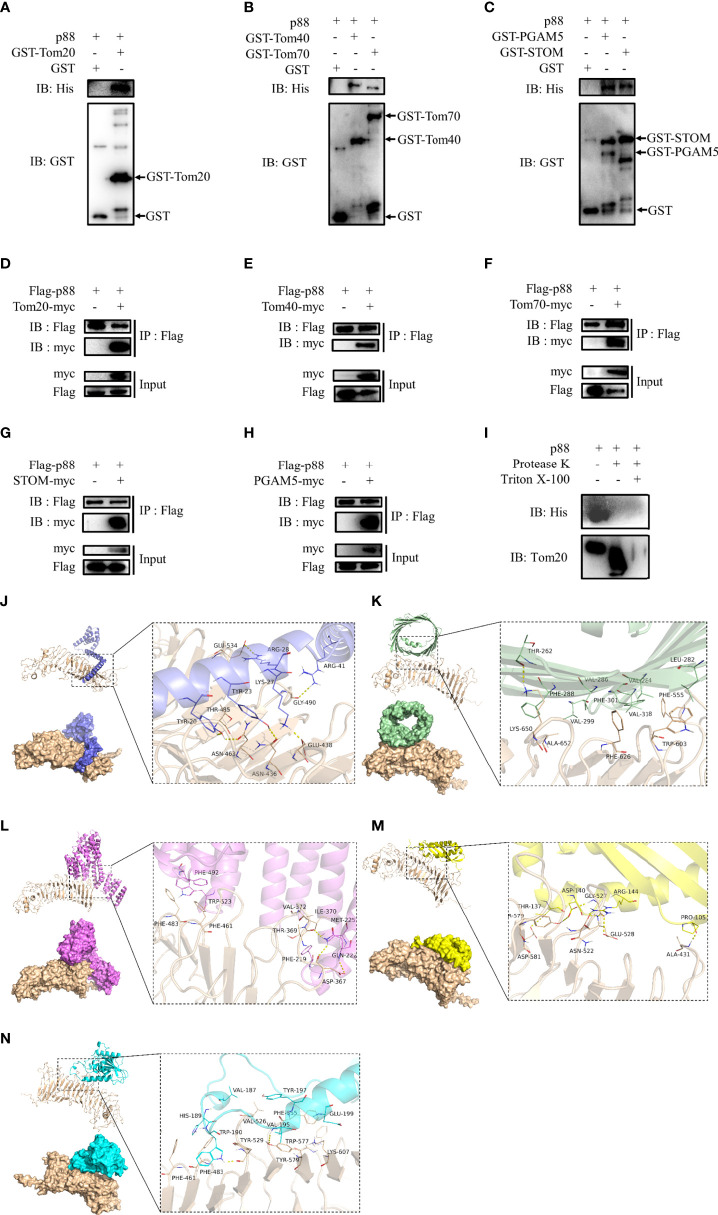
Proteins interacting with VacA. The combination of GST pull-down and western blot was used to detect interactions of Tom20 **(A)**, Tom40 **(B)**, Tom70 **(B)**, STOM **(C)**, and PGAM5 **(C)** with p88 (VacA). Co-immunoprecipitation experiments were carried out to further verify these interactions **(D-H)**. Protease K assays were performed to determine interactions of Tom20 and p88 **(I)**. Tom20 **(J)**, Tom40 **(K)**, Tom70 **(L)**, STOM **(M)**, and PGAM5 **(N)** were imported into ZDOCK to simulate possible combinations.

Considering that the entry of VacA into the inner membrane of mitochondria may require the participation of endocytosis of cell membrane, membrane vesicle transport, the translocase of outer mitochondrial membrane (TOM), and the translocase of inner mitochondrial membrane (TIM), we selected certain proteins (TMED9, HSP90AB1, phosphoglycerate mutase 5 (PGAM5), and stomatin (STOM)) or mitochondrial membrane proteins (Tom20, Tom22, Tom40, Tom70, VDAC1, VDAC2, VDAC3, Tim17, Tim21, Tim22, Tim23, and Tim50) for further research. To verify their interaction with VacA *in vitro*, the assay of GST pull-down was performed. Only the interactions of Tom20 (**Figure 8A**), Tom40 (**Figure 8B**), Tom70 (**Figure 8B**), STOM (**Figure 8C**), and PGAM5 (**Figure 8C**) between p88 (VacA) could be detected by western blot. To further determine the interactions between p88 and these proteins in the mitochondria, we isolated mitochondria from GES-1 cells. Taking Tom20 as an example, the results of the protease K assay showed that p88 was digested during Tom20 digestion (**Figure 8I**). These interactions have also been confirmed by co-immunoprecipitation (**Figures 8D–H**). The potential interaction sites of Tom20 (**Figure 8J**), Tom40 (**Figure 8K**), Tom70 (**Figure 8L**), STOM (**Figure 8M**), and PGAM5 (**Figure 8N**) with VacA (**Table S9**) have been predicted *via* protein-protein dockings by ZDOCK.

These results above indicate that the process of VacA entering mitochondria may require the binding of STOM to the cell membrane first. Then, VacA is transported into the mitochondrial membrane space and inserted into the inner mitochondrial membrane in the presence of interactions with TOM complexes (Tom20, Tom40, and Tom70) and PGAM5.

## Discussion

In this study, we demonstrated that brief exposure (24 h) of *Helicobacter pylori* (*H. pylori*) or *H. pylori* culture filtrate (HPCF) to GES-1 cells could induce autophagy. Our major findings include the following (1): VacA disrupted the mitochondrial membrane potential to induce PINK1/Parkin-dependent mitophagy in GES-1 cells (2). Cell membrane protein stomatin (STOM), inner mitochondrial membrane (IMM) protein phosphoglycerate mutase 5 (PGAM5), and outer mitochondrial membrane (OMM) proteins (Tom20, Tom40, and Tom70) might involve in this process.

It is generally believed that *H. pylori* infection is inextricably related to autophagy. *H. pylori* (22, 35–39) or its secretion may promote apoptosis, inhibit proliferation and induce autophagy of cells (40), which has been confirmed by our results (**Figures 1**, **2**). When we co-cultured gastric cell line GES-1 with *H. pylori* or HPCF for 24 h, the conversion of LC3B-I to LC3B-II was enhanced (**Figures 2C, D** **)**, and autolysosomes were formed (**Figure 2E**). The mechanisms underlying autophagy induction by *H. pylori* or HPCF are complex (21). Two virulence factors, vacuolating cytotoxin (VacA) and cytotoxin-associated gene A (CagA), play very important roles in autophagy (21–23, 34, 41, 42). Considering plenty of studies have shown that VacA can damage mitochondria and destroy the mitochondrial membrane potential (MMP), the first event we wanted to know was whether mitophagy is involved (43, 44). Although it is speculated that VacA can induce mitophagy (45), there is still a lack of evidence in this regard.

Subsequently, we explored the PINK1/Parkin-dependent mitophagy in *H. pylori*-treated or HPCF-treated GES-1 cells. Unfortunately, the expected changes in mitophagy-related protein levels did not occur (**Figure 2F**). This ‘confused mitophagy’ seems to contradict our existing cognition. We suspected that HPCF contains other components involved in the interference. However, HPCF led to changes in the expression level of these mitophagy-related proteins, which gave us some enlightenment. That is, some factors in HPCF might be involved in this effect on mitochondria. Among these virulence factors secreted by *H. pylori*, VacA damaging mitochondria is already widely studied.

To further evaluate the potential role of VacA (p88) in inducing autophagy of GES-1 cells, flow cytometry was used to detect apoptosis, proliferation (**Figure 4**), and the MMP *in vitro* (**Figures 5A, B**). The results of promoting apoptosis and inhibiting proliferation were consistent with those of *H. pylori* or HPCF. Generally, mitophagy can be further induced by the damage of mitochondria or the destruction of MMP. Changes in the expression level of mitophagy-related proteins in the PINK1/Parkin pathway confirmed that VacA (p88) could induce mitophagy *in vitro* (**Figure 6**), which confirmed our hypothesis.

**Figure 9:**
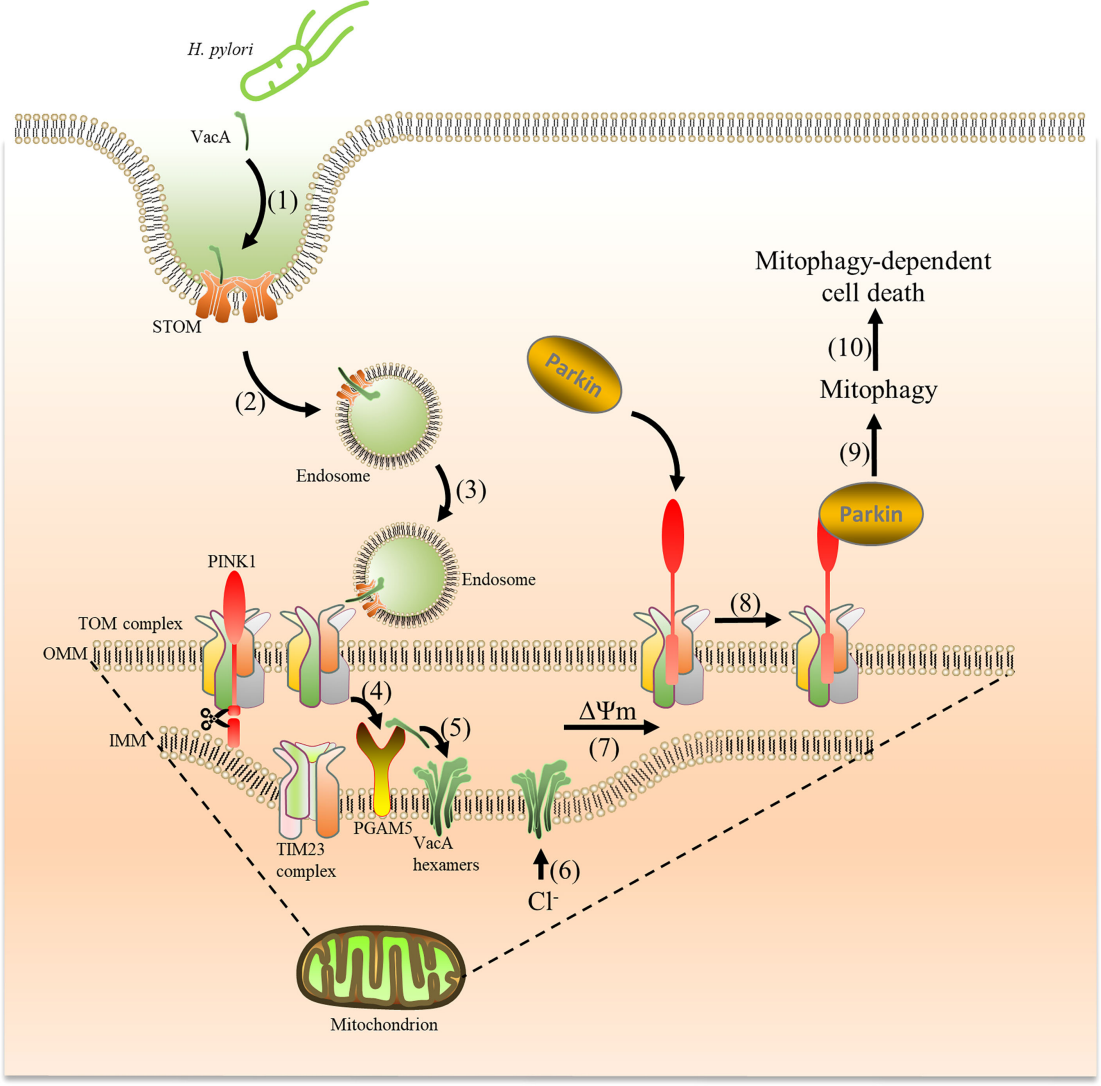
VacA secreted by *H. pylori* is captured by cells and targets mitochondria, which induces mitophagy. Acid-activated VacA is first captured by STOM on the cell membrane (1), and then forms endosomes to enter cells (2) and target mitochondria (3). VacA binds to the TOM complex (translocase of the outer membrane) and is transported into the mitochondrial membrane space (4). Subsequently, PGAM5 (on the inner mitochondrial membrane) binds to VacA and aids in inserting VacA into the inner mitochondrial membrane (5) to form Cl^−^ channels (6). The imbalance of Cl^-^ reduces the membrane potential (7), promoting PINK1 accumulation and Parkin recruitment from the cytosol to mitochondria to induce mitophagy (8) (9),. Mitophagy eventually leads to cell death (10).

The proteins are involved at the heart of our understanding of VacA entering cells and inducing mitophagy. The binding of VacA to the surface of eukaryotic cells is the first step in cell poisoning. The first cell surface protein considered the receptor for VacA is p140 (46). The EGF receptor (47) and RPTPbeta (48) are also considered in combination with VacA. Our results demonstrate that STOM can also be combined with VacA. Previous studies have shown that this 31 kDa-integrated membrane protein is involved in various physiological processes, including membrane fusion (49), anion exchange (50), and material transportation (51). Therefore, we have reason to believe that VacA is internalized through endocytosis by binding to STOM of the outer membrane of host cells, forming early endosomes (**Figures 8C, G**, **9**), and targeting mitochondria (**Figure 9**) (12). Some studies have shown that Tom20 and Tom40 of OMM are involved in the transport of VacA into mitochondria (52, 53). Our experimental results show that Tom20, Tom40, and Tom70 of the TOM complex are all involved in the transport of VacA (**Figures 8A, B, D–F**). Unfortunately, we did not detect the interaction between other OMM proteins (Tom22, VDAC1, VDAC2, and VDAC3) and VacA *in vitro*.

When VacA is transported through the TOM complex of OMM, it enters the mitochondrial membrane gap and then forms an anion channel in the inner membrane of the mitochondrial (12). Although we did not find evidence that the TIM complex (Tim17, Tim21, Tim22, Tim23, and Tim50) of IMM binds to VacA, PGAM5 seems to be involved (**Figures 8C, H**). PGAM5 is closely related to mitochondrial damage (20) and is located in the IMM, which has been confirmed by previous studies. The anion channel formed by VacA is conducive to the transport of Cl^-^. The imbalance of Cl^−^ causes depolarization of mitochondria, retards the import of PINK1, which leads to the instability of PINK1 on the damaged mitochondria and evokes PINK1/Parkin-dependent mitophagy.

The results of acid-activated VacA inducing mitophagy made us realize that acid-activation is so important for the display of VacA activity (54–56). In our pre-experiment, we found that non-acid-activated VacA can also induce the formation of puncta in HeLa YFP-Parkin cells. But, the expression levels (similar to that of *H. pylori* or HPCF) of mitophagy-related genes did not indicate the occurrence of mitophagy. This suggests that acid-activation is necessary for VacA to induce true mitophagy. Although we have verified the interaction between these membrane proteins (STOM, Tom20, Tom40, Tom70, and PGAM5) and VacA *in vitro*, we still lack evidence *in vivo*. We indirectly verified the interaction between Tom20 and VacA through a protease K protection assay. However, these interactions of acid-activated p88(VacA) with mitochondrial proteins could not be directly affirmed in GES-1 cells or mitochondria by co-immunoprecipitation. Accordingly, we overexpressed VacA and the above mitochondrial membrane proteins in cells, and the physical interactions between them have successfully been confirmed by *in vivo* co-immunoprecipitation. These are helpful for us to understand how VacA enters the mitochondria and induces mitophagy. Without a doubt, some deficiencies still existed in our experiment. Next, the interaction between VacA and mitochondrial proteins will be studied under real *in vivo* physiological conditions to further elucidate the process of VacA entering mitochondria and the detailed mechanisms of triggering mitophagy by VacA in GES-1 cells.

## Conclusions

In conclusion, we determined that *H. pylori*-derived VacA induced mitophagy through the PINK1/Parkin pathway in gastric mucosa epithelial cells. The cell membrane proteins STOM, TOM complex (Tom20, Tom40, and Tom70), and PGAM5 from IMM may play important roles in facilitating transport and completing the process. These findings described here provide the framework for ongoing studies to understand how VacA induces mitophagy and injures gastric epithelial cells.

## Publisher’s Note

All claims expressed in this article are solely those of the authors and do not necessarily represent those of their affiliated organizations, or those of the publisher, the editors and the reviewers. Any product that may be evaluated in this article, or claim that may be made by its manufacturer, is not guaranteed or endorsed by the publisher.

## Data Availability Statement

The original contributions presented in the study are included in the article/**Supplementary Material**. Further inquiries can be directed to the corresponding authors.

## Author Contributions

Conceptualization, LW, JY, and H-LW. Methodology, LW and JY. Soft-ware, J-XH and GC. Validation, L-NW, X-YY, and X-YW. Formal analysis, BX. Investigation, JC. Resources, JY. Data curation, Q-FW. Writing—original draft preparation, LW. Writing—review and editing, LW, JY, and H-LW. Visualization, LW. Supervision, JY and H-LW. Project administration, LW, JY, and H-LW. Funding acquisition, JS, L-MH, and TJC. All authors listed have made a substantial, direct, and intellectual contribution to the work and approved it for publication.

## Funding

This work was supported by the Natural Science Foundation of Gansu Province, China (Nos. 20JR5RA281 and 20JR5RA354), the Open Project of Key Laboratory of Functional Genomics and Molecular Diagnosis of Gansu Province (No. 2020BYGT-001), the Fundamental Research Funds for the Central Universities (No. lzujbky-2021-it19), the Open Project of State Key Laboratory of Bioactive Substance and Function of Natural Medicines, Institute of Materia Medica, Chinese Academy of Medical Sciences and Peking Union Medical Colleges (No.GTZK202006), and the Project of Cuiying Technological Innovation of Lanzhou University Second Hospital.

## Conflict of Interest

Authors T-JC and H-LW were employed by Lanzhou Baiyuan Gene Technology Co., Ltd.

The remaining authors declare that the research was conducted in the absence of any commercial or financial relationships that could be construed as a potential conflict of interest.

## Publisher’s Note

All claims expressed in this article are solely those of the authors and do not necessarily represent those of their affiliated organizations, or those of the publisher, the editors and the reviewers. Any product that may be evaluated in this article, or claim that may be made by its manufacturer, is not guaranteed or endorsed by the publisher.
