# Supplementary material for: Vacuolating Cytotoxin A Triggers Mitophagy in Helicobacter pylori-Infected Human Gastric Epithelium Cells
Source: Front Oncol. 2022 Jul 14;12:881829. doi: 10.3389/fonc.2022.881829 (PMC9329568; doi:10.3389/fonc.2022.881829)
Supplement: Supplementary file 2 [file DataSheet_2.docx]

Supplementary Material

# Supplementary Figures


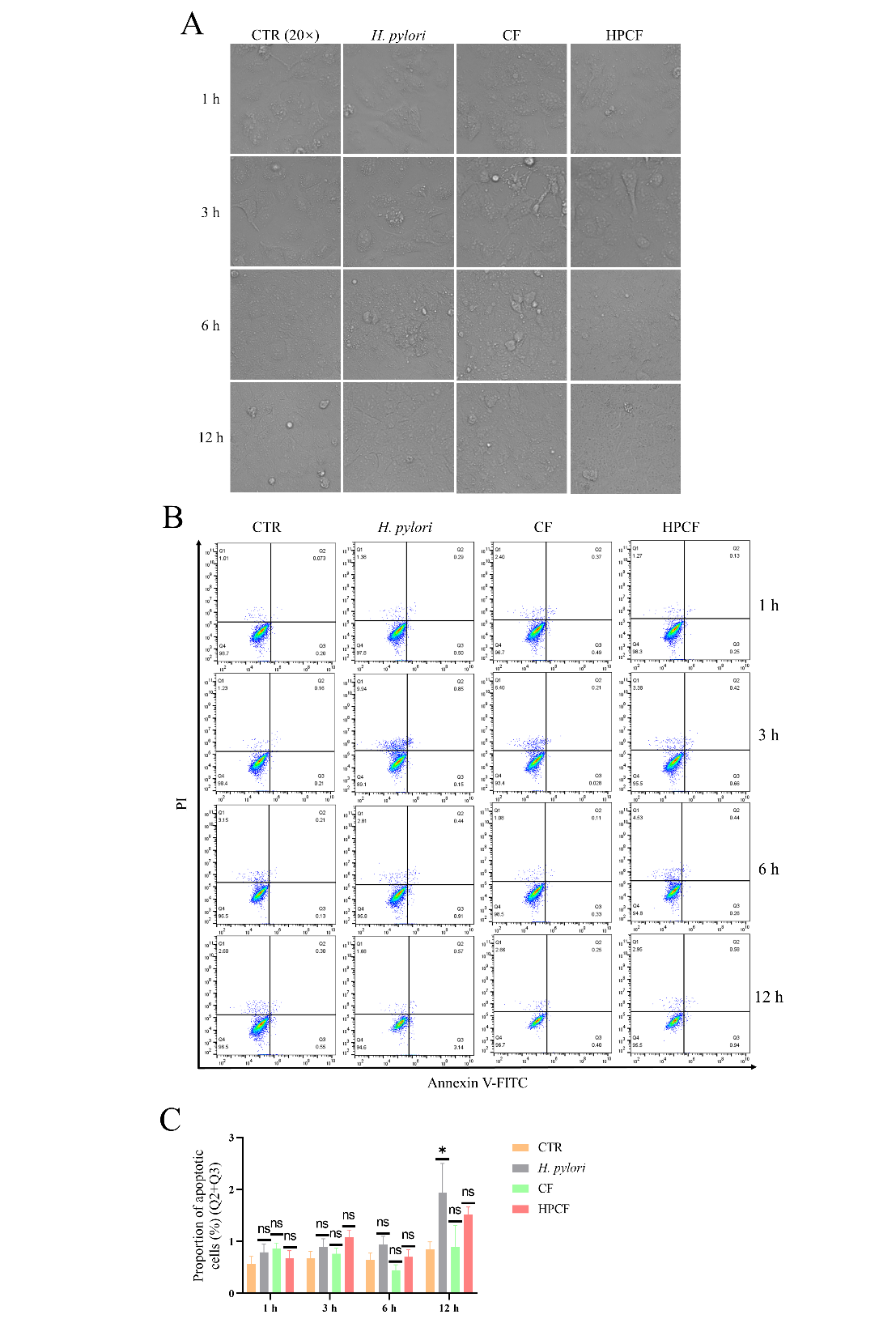


**Figure S1**. The time course of *H. pylori*-induced GES-1 cells apoptosis. Representative apoptosis photographs of GES-1 cells treated with *H. pylori* **(A)**. The apoptotic GES-1 cells were detected by flow cytometry **(B)**. Quantitative analysis of apoptosis (Q2+Q3) of GES-1 cells treated with *H. pylori* was performed **(C)**. The asterisks indicate significant differences from the control. n=3. **p* < 0.05. ns, no significance.


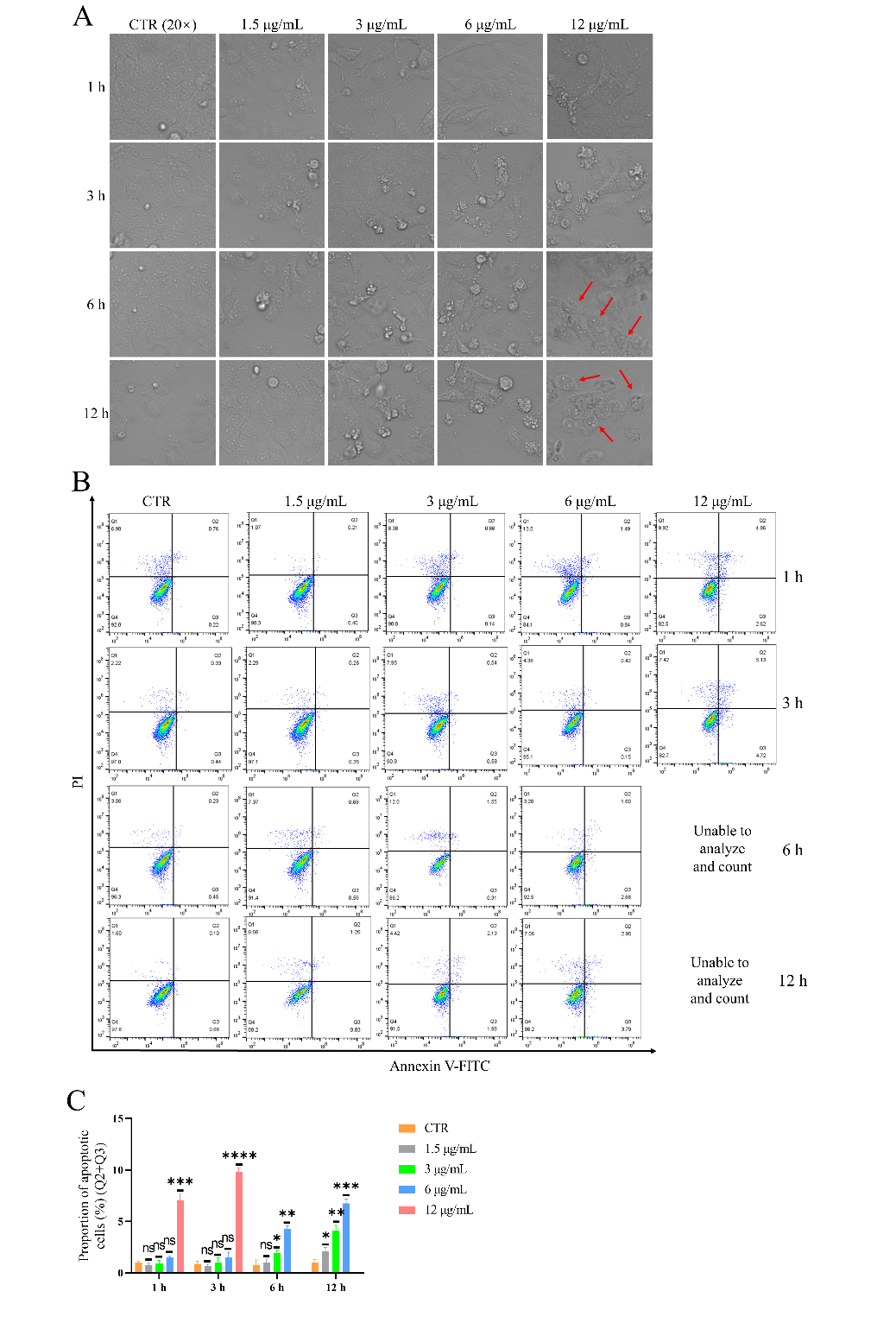


**Figure S2**. The time course of p88-induced GES-1 cells apoptosis. Representative apoptosis photographs of GES-1 cells treated with p88 **(A)**. The apoptotic GES-1 cells were detected by flow cytometry **(B)**. Quantitative analysis of apoptosis (Q2+Q3) of GES-1 cells treated with p88 was performed **(C)**. The asterisks indicate significant differences from the control. n=3. **p* < 0.05, ***p* < 0.01, ****p* < 0.001, *****p* < 0.0001. ns, no significance.


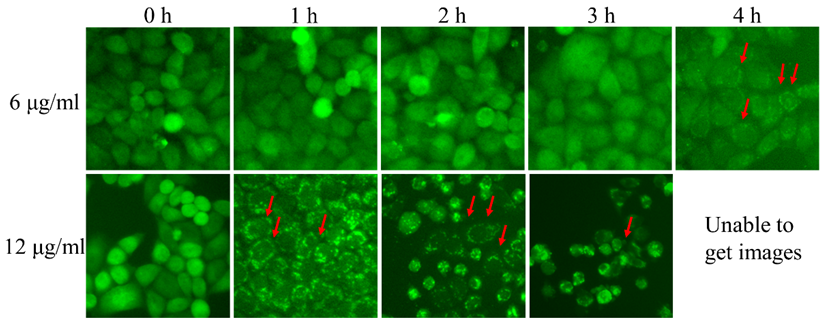


**Figure S3**. The translocation of Parkin induced by p88 in HeLa YFP-Parkin cells. After being treated with acid-activated p88 (6 μg/mL or 12 μg/mL), the representative fluorescence images (20×) of the formation of Parkin puncta.


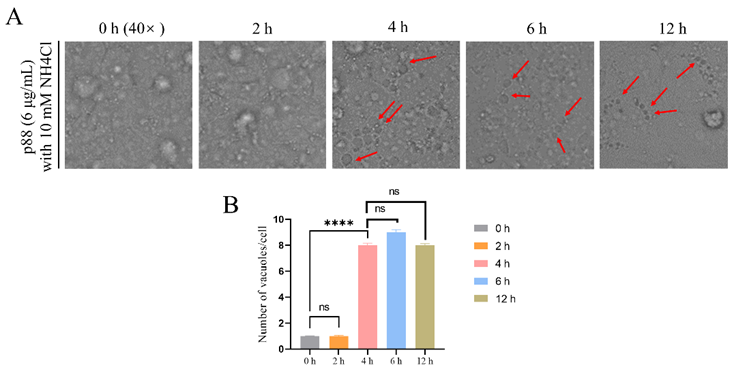


**Figure S4**. The time course of vacuolar denaturation. After being treated with acid-activated p88 (6 μg/mL), The number of vacuoles formed at different time points is counted **(A, B)**. The asterisks indicate significant differences from the control. n=3. *****p* < 0.0001. ns, no significance.
